# The prevalence of occupational injuries and associated risk factors among workers in iron and steel industries: a systematic review and meta-analysis

**DOI:** 10.1186/s12889-024-20111-w

**Published:** 2024-09-27

**Authors:** Saumu Shabani, Jovine Bachwenkizi, Simon Henry Mamuya, Bente Elisabeth Moen

**Affiliations:** 1 Department of Environmental and Occupational Health, School of Public Health and Social Sciences, Muhimbili University of Health and Allied Sciences, Dar-es-Salaam, Tanzania; 2 Department of Global Public Health and Primary Care, Centre for International Health, University of Bergen, Bergen, 5020 Norway

**Keywords:** Occupational injury, Pooled prevalence, Systematic review, Meta-analysis

## Abstract

**Background:**

The iron and steel industries are among the most dangerous workplaces in the world compared to other manufacturing industries. Workers are exposed to multiple occupational hazards, which predispose them to high risks of both fatal and non-fatal injuries. Currently, the data on the global prevalence and associated risk factors for occupational injuries in the iron and steel industries is fragmented and incomplete. This study was undertaken to address this issue by pooling data relating to the prevalence of occupational injuries and its associated factors among workers in iron and steel industries studies around the world.

**Methods:**

The search was conducted systematically using PubMed, HINARI, EMBASE and Google Scholar for published studies in English that reported on occupational injuries and associated risk factors among workers in iron and steel industries. MetaXL version 5.3 software was used in the meta-analysis to estimate the pooled prevalence of occupational injuries and associated risk factors among workers in the iron and steel industries. The study protocol has been registered with PROSPERO, number CRD42022344258.

**Results:**

Of the 447 articles identified, 15 studies from 9 countries met the inclusion criteria. The pooled prevalence estimate of occupational injury was 0.55 (95% CI: 0.15, 0.93). The pooled results indicated that the odds of having an occupational injury were 4.06 times higher among workers who did not use personal protective equipment compared to those who used such equipment. Likewise the odds of occupational injuries was increased by 1.65 among night shift workers compared to the counterpart.

**Conclusions:**

The global prevalence of occupational injuries in iron and steel industries was 55%. The results indicate that night work shift and the lack of use of personal protective equipment has a higher impact than other factors in the occurrence of occupational injuries in the iron and steel industries.

**Supplementary Information:**

The online version contains supplementary material available at 10.1186/s12889-024-20111-w.

## Background

Occupational injuries are a global public health problem and might be a symptom of poor safety management in workplaces. According to the International Labour Organization (ILO), an occupational injury is defined as any personal injury, disease or death resulting from an occupational accident [1]. An occupational accident is defined as an unexpected accident arising out of or in connection with work which results in one or more workers incurring a personal injury, disease or death [1]. Occupational injuries tend to raise costs directly and indirectly for individuals, societies, and nations and have a negative impact on workplaces due to increased staff turnover and increased absenteeism, which necessitates more training, decreased production, as well as higher insurance and workers' compensation premiums [2]. Occupational injuries tend to be more severe in low- and middle-income countries (LMICs) [3], where workplace safety often is neglected.

Iron and steel industries are industries producing the metal steel. Steel is an alloy of iron and carbon [4]. Steel production involves separating metals from iron ores and/or scrap and subsequently refining the metal to more pure forms. The steelmaking has two stages. First, the reduction stage, i.e. ironmaking stage (iron ore reduced to hot metal), and second the oxidation stage, i.e. steelmaking stage (hot metal refined to steel) [4]. The processes also includes work at furnaces. This production has been increasing in many countries the past years, as the iron and stel industry is very important in global manufacturing and construction. The sector plays an important role in the production of essential materials for e.g. automobiles, technical innovations and infrastructure, and is important for the development of low-and middle-income countries. However, the iron and steel industries are among the most dangerous workplaces in the world compared to other manufacturing industries [5, 6]. The workers in iron and steel industries are exposed to several health hazards during the steel making processes. The workers might experience burns, fractures and sprains due to slips, trips and falls, as well as fatalities. In 2005, the International Labor Organization (ILO) published a report on the code of practice on safety and health in the iron and steel industry that detailed the most common causes of injuries and illnesses in the iron and steel industry and suggested strategies and practical guidance for the management of workplace hazards [7]. However, the rate of occupational injuries in iron and steel industries has not decreased since this time and might even have increased in some countries [8–11]. No studies on occupational injuries have been undertaken in the iron and steel industries worldwide, only a few studies in separate countries. The existing body of literature is fragmented, and it is difficult to know the real magnitude of the problem globally. For example, in Ethiopia, there are four studies conducted in iron and steel industries [9, 12–14]; in India, four studies have been conducted [5, 15–17]; in Iran, three studies[18–20]; in Turkey [21] and Brazil two studies [11, 22]. Poland [23], Saud Arabia [24], Sweden [25], and Singapore [26]. The injury prevalence from these studies varies from 12% in Iran to 72.7% in Brazil.

Many of the studies concerning occupational injuries in the iron and steel industry mention different risk factors that might be associated with occupational injuries. The most common risk factors mentioned in this industry include the level of education [18], work experience [12], PPE utilization [13], shiftwork (24), working hours [12], work stress [27], sex [9], age [16], safety and health supervision [12], and OHS training [14].

To effectively interpret data relating to the global prevalence of occupational injuries based on specific industries is important. Studies which combine data about global occupational injuries and their associated factors of specific industries can help to demonstrate whether OHS interventions work in both developed and developing countries. They will also provide information about the prominent risk factors that contribute to occupational injuries. The findings will help the policy makers, employers, and workers to work together to develop effective strategies to prevent and manage occupational injuries, promote worker safety and health, and improve the overall working conditions. In our study, we focus on one specific industry, the iron and steel industry, which involves iron and steel mills, ferroalloy manufacturing, steel product manufacturing from purchased steel, foundries, and iron and steel forging. Our aim of the study was to produce a review of the prevalence of occupational injuries and their associated risk factors among workers in this industry, including studies from countries world-wide. The review was guided by the research question: "What are the pooled prevalence and associated risk factors of occupational injuries among workers in iron and steel industries in a global perspective?". We wanted to study the prevalence of occupational injuries in different regions of the world and also related to the classification of the country income [28].

## Methods

### Searching strategies

We searched articles from the following databases: PubMed, HINARI, EMBASE, Google Scholar and citation search by using the following keywords: (occupational injury]) OR (occupational accident) OR (work-related injury) OR (work-related accident) OR (workplace injury) OR (workplace accident) OR (fatal injury) OR (non-fatal injury) AND (risk factor) OR (factors) OR (factors associated) OR (predictors) AND (iron industry) OR (steel industry) (metal industry) OR (metallurgical industry) OR (foundry industry). The key terms were combined using Boolean operators "OR" and "AND" (Additional file 1).

### The inclusion criteria

The review considered all scientific studies in international journals from the iron and steel industries that reported occupational injuries. The outcome of the studies should be occupational injuries among the employed workers, with or without information about associated risk factors. We defined an occupational injury as an unexpected and unplanned event that has occurred in the workplace while a worker is fulfilling their duties, leading to injury, illness, or death and restrict from working at least one day after the injury occurred. Iron and steel industries were included if they were primarily engaged in one or more of the following: (1) direct reduction of iron ore; (2) manufacturing pig iron in molten or solid form; (3) converting pig iron into steel; (4) making steel; (5) making steel and manufacturing shapes (e.g., bar, plate, rod, sheet, strip, wire); (6) making steel and forming pipe and tube; and (7) manufacturing electrometallurgical ferroalloys. The study designs included were epidemiological studies (cohort, case-control and cross-sectional (observational) studies), and the studies should be in English language and published from 1972/1/1 to 2022/12/31. The starting year was chosen as we knew there was a study from the industry this year.

### The exclusion criteria

The review excluded all studies conducted on workers from industries other than iron and steel industries, case studies, studies using qualitative research methods, and randomized controlled trials. Moreover, studies with low quality were excluded, using the pre-settled parameters according to The Joanna Briggs Institute critical appraisal checklist for reviews of prevalence studies (Additional file 2). Duplicates were removed, and the database search results were merged using Mendeley software. The website of the index of diverse open access journals (DOAJ) was used to check the studies. If the journal was not listed there, it was anticipated to be a predatory journal, and these studies were excluded. Grey literature (reports) was not included.

### Study selection and data extraction process

Two authors assessed the initially selected abstracts independently against the stated study inclusion criteria. This was followed by an assessment of the full papers of the potential studies. Data were defined and extracted. The data extracted included the first author's name, publication year, title, country, study design, group of workers studied, number of industries included, type of data, sample size, sex, mean age, prevalence, type of injury, and response rate.

### Associated risk factors of occupational injuries

All eligible articles were screened to identify the studies that investigated particular risk factors for occupational injuries. The associated risk factors assessed in this review were sex, age group, education level, marital status, safety training, use of PPE, working hours, and shift work.

### Assessment of study quality and risk of bias

The Joanna Briggs Institute critical appraisal checklist for reviews of prevalence studies with 9 criteria [29], was used in this study. Each criterion was given either of the two options of YES if a criterion was met or NO if a criterion was not met. A YES option was graded as 1, and a NO option as 0. A minimum score of 0 was given if all criteria were not met, and a maximum score of 9 was given if all criteria were met. Studies with overall grades ranging from 0–4 were considered of low quality, 5–7 moderate quality and 8–9 high quality. Studies with moderate to high quality were included in the review. Two reviewers (SS and JB) independently assessed the quality of selected articles.

### Data analysis

Data were stored in a predefined spreadsheet file, including the authors' names and year of publication, the number of workers included in the studies, and the number of injured workers. Data were analyzed using Metal version 5.3 software, an add-in for meta-analysis in Microsoft Excel for Windows (https://www.epigear.com/indexfiles/metaxl.html). The pooled prevalence and odds ratio were synthesized by using the quality effect (QE) model with the double arcsine method. Heterogeneity between estimates was assessed using the forest plots and I-squared statisticI-squared statistics < 25% indicated a low level, 25–50% indicated a moderate level, and > 50% indicated a high level of heterogeneity. Potential risk factors influencing the prevalence estimates were investigated using subgroup analyses. Subgroup analyses were performed based on regions used by the World Health Organization. In addition, sub analyses were made based on the income levels of the countries, using the World Bank country classifications. Also, analyses were made based on the data type (self-reported and recorded data) to understand the effects on the prevalence of occupational injuries. We also performed sensitivity analyses by deducting each study and calculating the aggregated prevalence rate for the remaining studies to identify the source of heterogeneity further. A Doi plot and Luis Furuya-Kanamori (LFK) index were used to detect the likelihood of publication bias. These are the best tools for capturing the publication bias in the smallest studies with either 5, 10, or 20 included studies. An LFK index within ± 1, out of ± 1 but within ± 2, and > ± 2 refer to no asymmetry, minor asymmetry, and major asymmetry, respectively [30].

## Results

**Fig. 1:**
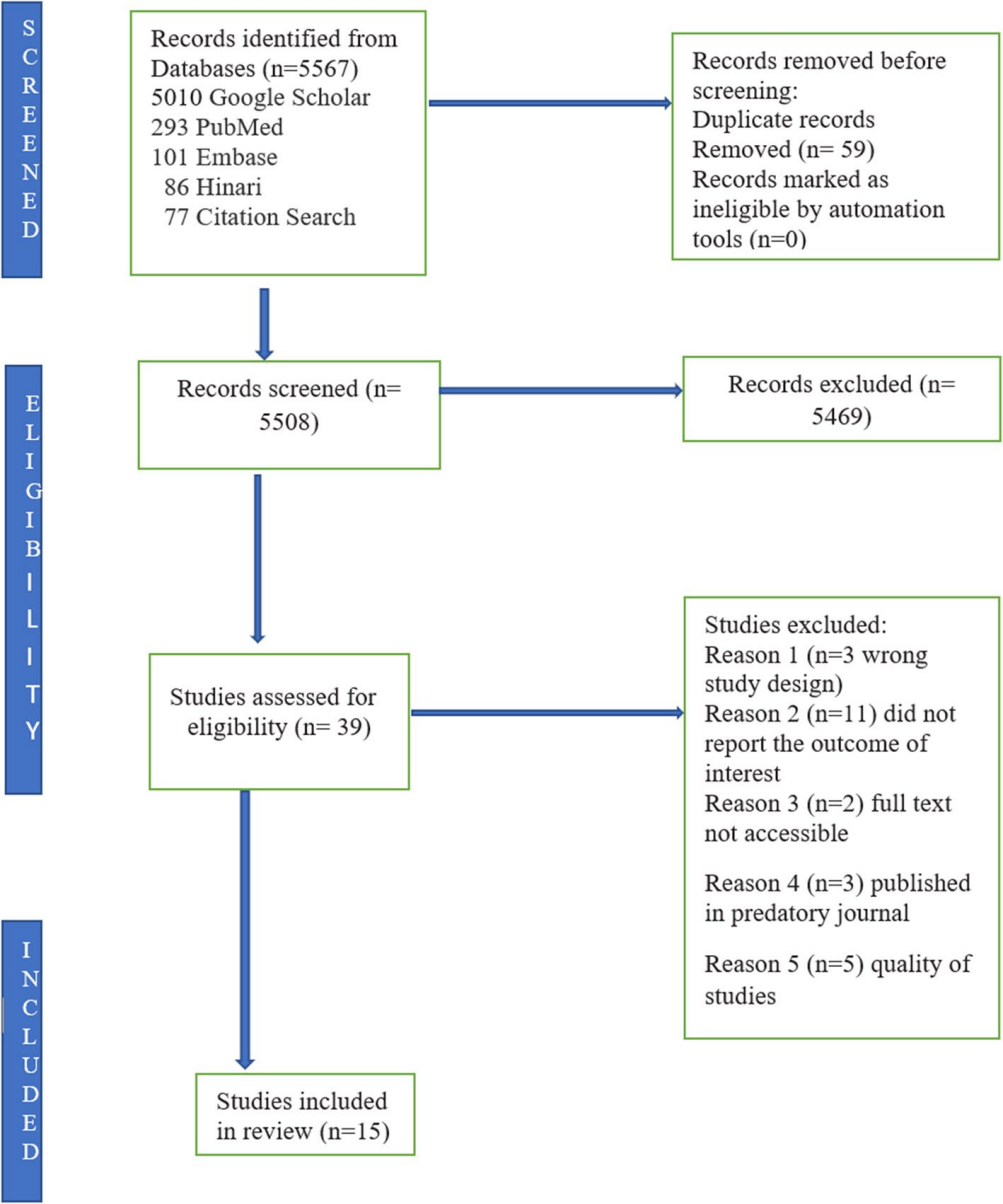
The PRISMA flow diagram for article searching and selection process of studies

The search resulted in a total of 447 articles. After the removal of duplicates, only 398 potentially relevant journal articles remained. Of these remaining articles, 359 were excluded at the title and abstract screening level, leaving only 39 full-text articles to be later assessed for eligibility. Fifteen studies met the inclusion criteria and hence were used for this study (Fig. 1).

### Characteristics of included studies

Studies included in the synthesis 14 were cross-sectional studies [8, 10–15, 18–22, 24, 27, 32] and one cohort study [11]. Among the included studies, four were from Africa; three were from the Americas, three were from the European region; three from the Eastern Mediterranean region, two from the South East Asia region, and 1 from the Western Pacific region. A total of 36470 study participants were involved in all the included studies combined. Details of the included studies are presented in Table 1.

**Table 1:** Characteristics of included studies in a systematic review and meta-analysis of the prevalence of occupational injuries and associated risk factors among workers in iron and steel industries

Study 1st author	Reference	Year	Country	WHO Region	World Bank classifi-cation	Study Design	Study type	Response rate	Sample size
Asadi Z.	[31]	2018	Iran	Eastern Mediterranean Region	LMICs	CS	Recorded injuries	na	178
Ballal S.G	[24]	1997	Saudi- Arabia	Eastern Mediterranean Region	HICs	CS	Recorded injuries	na	689
Benti A.	[32]	2019	Ethiopia	African Region	LICs	CS	Self-reported	99	583
Berhan E.	[9]	2020	Ethiopia	African Region	LICs	CS	Self-reported	100	446
Bylund PO	[25]	1998	Sweden	European Region	HICs	CS	Recorded injuries	na	2156
Gonçalves SB.	[22]	2018	Brazil	Americas	UMICs	CS	Recorded injuries	na	1,277
Gulhan B.	[21]	2012	Turkey	European	UMICs	CS	Self-reported	95.7%	201
Kilfe M.	[13]	2013	Ethiopia	African Region	LICs	CS	Self-reported	98	453
Kumar S.G.	[16]	2014	India	South Eastern Asia Region	LMICs	CS	Self-reported	Na	209
Mazaheri MA.	[20]	2009	Iran	Eastern Mediterranean Region	LMICs	CS	Recorded injuries	Na	5734
Ogiński A.	[23]	2015	Poland	European	HICs	CS	Recorded injuries	Na	1200
Ong CN.	[26]	1987	Singapore	Western Pacific Region	HICs	CS	Recorded injuries	Na	664
Rajak R.	[17]	2021	India	South Eastern Asia Region	LMICs	CS	Self-reported	Na	505
Schoemaker MJ	[11]	2000	Brazil	Americas	UMICs	Cohort	Recorded injuries	Na	21732
Sime A.	[14]	2020	Ethiopia	African Region	LICs	CS	Self-reported	100	443

*na * not available, *CS *Cross sectional

### Study quality and risk of bias assessment results

The study quality and risk of bias for selected studies for systematic review and meta-analysis were based on 9 criteria as described in the Joanna Briggs Institute critical appraisal checklist for use in reviews of prevalence studies. The results showed 5 studies with low quality (score 0–4), which were removed from meta-analysis. Fourteen studies scored moderate quality (5–7), and 1 study scored high quality (8). The average score was 5.75, which indicates the overall moderate quality of the included studies (Additional file 2).

### The pooled prevalence of occupational injuries among workers in iron and steel industries

**Fig. 2:**
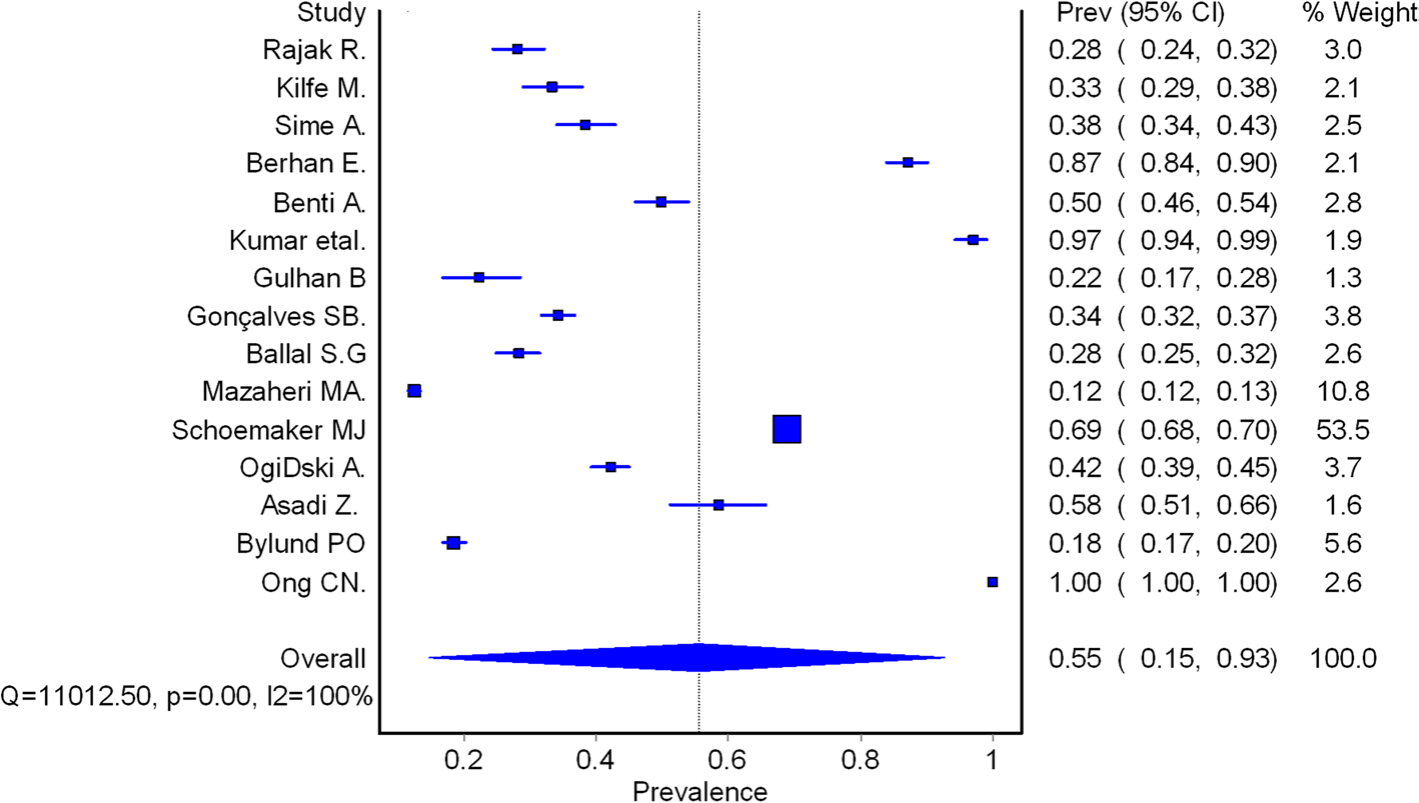
Pooled prevalence of occupational injuries among workers in iron and steel industries

A total of 36470 study participants from 15 studies were used to estimate a pooled prevalence of occupational injuries among workers in iron and steel industries (Fig. 2). This pooled prevalence of occupational injuries among the workers was 0.55 (95% CI: 0.15, 0.93). The degree of between-studies variances was substantially high, as indicated by I-squared statistics = 100%.

### Sub-group analyses

**Fig. 3:**
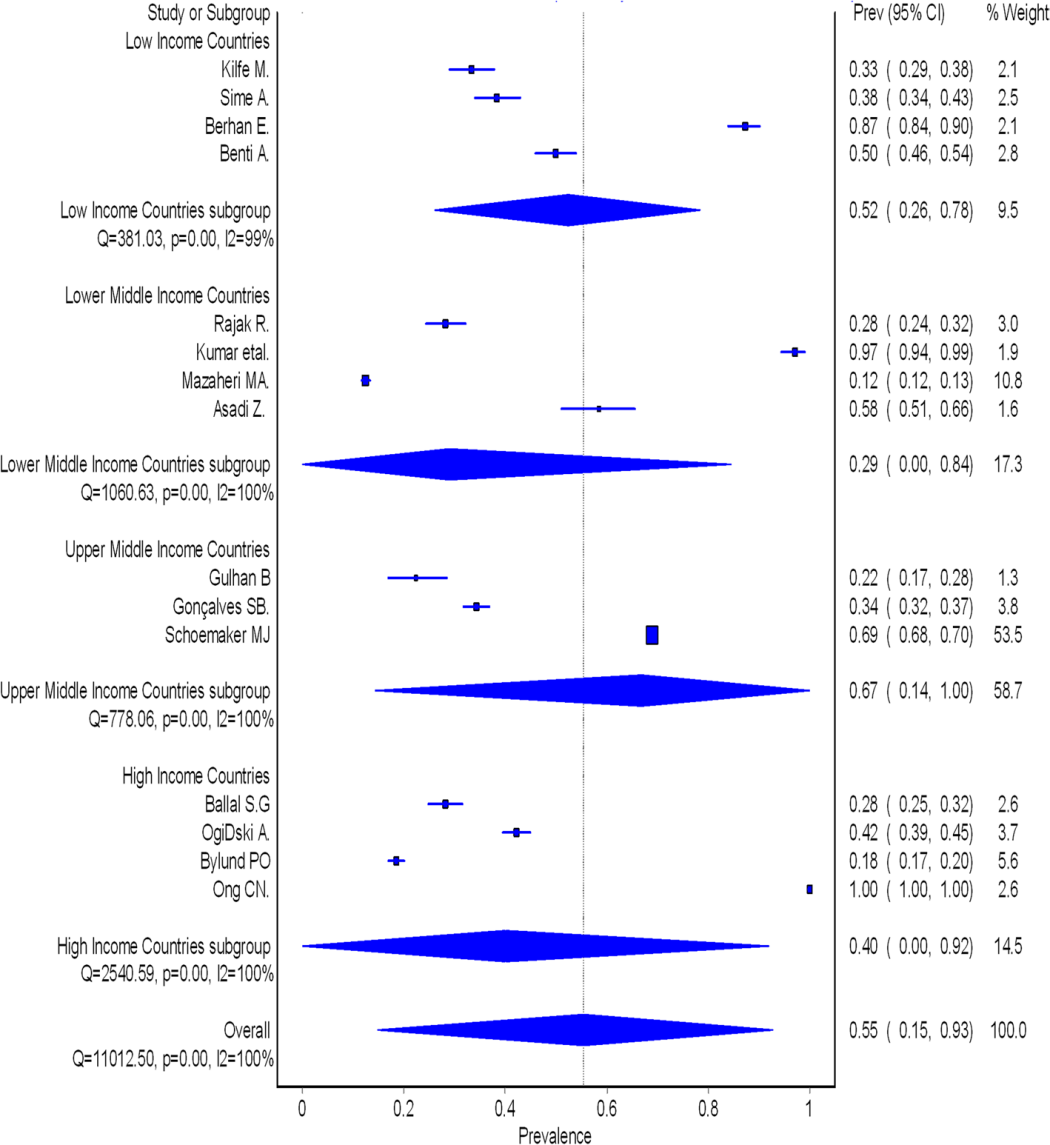
Forest plot of subgroup analysis of the pooled prevalence of occupational injuries based on the World Bank classification of the income of the countries

The pooled prevalence of occupational injuries was influenced by substantial heterogeneity; therefore, we performed some subgroup analyses based on the WHO regional locations for the study, World Bank classifications and data type (self-reported and recorded data). A significant association was observed for each of the variables. Still, for all of them, there continued to be heterogeneity ranging from 99–100%, suggesting that WHO regional classification, World Bank classification, and study design were not the cause for heterogeneity (Fig. 3 and Additional file 3).

### Risk factors associated with occupational injuries

**Fig. 4:**
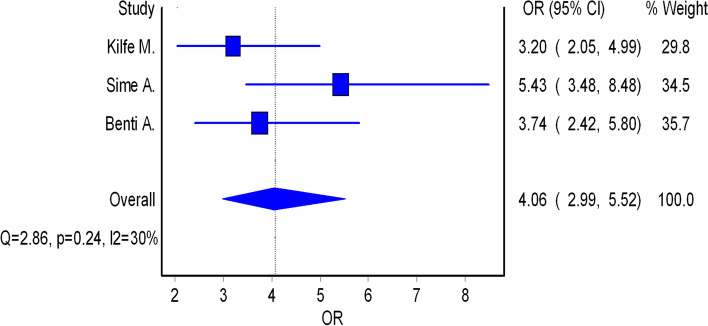
Forest plot of the odds ratios (OR) with corresponding 95% CIs of studies on the association of PPE use and occupational injuries

**Fig. 5:**
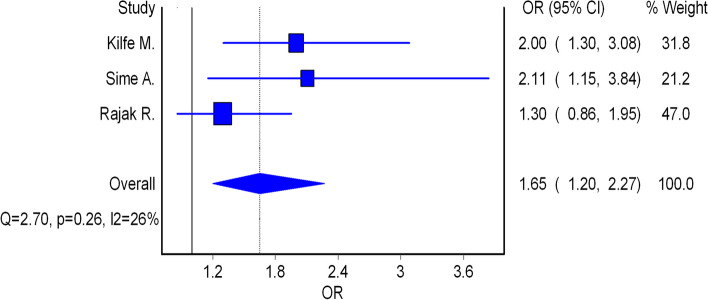
Forest plot of the odds ratios (OR) with corresponding 95% CIs of studies on the association of work shift and occupational injuries

Of the 15 studies, seven reported on risk factors for occupational injuries among workers in iron and steel industries [11–14, 17, 21, 31]. Three of the included studies reported an association between personal protective equipment (PPE) use and occupational injuries [12–14]. This association indicated that the odds of having occupational injury were higher among workers who did not use PPE compared to those who were using PPE. The pooled effect indicated that the odds of having occupational injury were 4.06 times higher among workers who did not use PPE compared to those who were using PPE (Fig. 4). Regarding shiftwork, the findings of this review showed that the odds of having an occupational injury among night shift workers were higher compared to those workers who did not have night shifts (Fig. 5).Furthermore, the pooled estimate also showed that the odds of having an occupational injury among workers who had night shiftwork were 1.65 times higher compared to those workers who did not have a night shifts (Fig. 5).

Other factors discussed in the included studies were marital status, education level, age and safety training. However, their pooled estimates were not statistically significant, as shown in supplementary information (Additional files 4–8).

### Publication bias

The Doi plots and LFK indices for pooled prevalence was LFK index − 0.76, no asymmetrical, PPE use was LFK index 0.64 no asymmetry and work shift was LFK index-0.73 no asymmetry, all these indicating the absence of publication bias.

### Sensitivity analysis

We performed sensitivity analyses, and their results showed that the pooled estimated prevalence of occupational injuries obtained when each of the included studies was left out from the analysis at a time was within the 95% confidence limit of the pooled estimate of occupational injuries when all studies were pooled together. This suggested that a single particular study did not influence the overall average prevalence. The average prevalence of occupational injuries when each of the 15 studies was left out from the analysis ranges between 0.357% (95% CI 0.150, 0.592) and 0.623 (95% CI 0.214, 0.960).

## Discussion

The findings show a pooled prevalence of occupational injuries among workers in the iron and steel industry is 0.55 (95% CI: 0.15, 0.93). This injury prevalence seems high, as almost more than half of the workers were injured. However, it is difficult to compare this prevalence figure with results from related previous studies, as there are no similar reviews from the iron and steel industry. To our knowledge, this is the first systematic review and meta-analysis that provides the pooled prevalence of occupational injuries among workers in iron and steel industries worldwide. However, the findings seem to be higher compared to the global prevalence of occupational injuries in general [33]. The difference in findings can be due to the methods used in incorporating the global injuries data. In contrast, the injury data are mainly obtained from reported cases at labour or OSHA offices. It is known that in many countries, especially developing countries and lower-middle-income countries, injury cases are under-reported, and the accuracy of data documentation is still a problem. Therefore, this situation tends to affect the global figure of the injuries. Also, the pool prevalence of occupational injury in this review is higher than a pooled prevalence of 0.44 found in a meta-analysis concerning occupational injuries in general in Ethiopia [34], and lower than the pool prevalence of occupational injury among workers in the construction, manufacturing, and mining industries in Africa [35]. The higher pooled prevalence of occupational injuries in iron and steel industries can be explained based on the overall process of metal manufacturing from the beginning up to the finishing part, where it involves a lot of hazardous substances, such as heat, noise, machines, vehicles, etc. Also, most of manufacturing industries, such as iron and steel industries, used to hire large numbers of workers with the following characteristics such as low skills, low socioeconomic status, young age, male gender, illegal immigrant status, lack of language proficiency, poor communication and lack of on-the-job training in order to pay them low and earn more profit. Hence, these characteristics of workers and the working environment contribute to the increased risk of occupational injuries in these industries [36].

The subgroup analyses showed a substantial variation in the pooled prevalence of occupational injuries in both WHO regions and in the World Bank classification. The region with the highest pooled prevalence of occupational injuries was the western Pacific region, followed by America and the African region, and the lowest pooled prevalence was in the Eastern Mediterranean region. This difference in the prevalence of occupational injuries can be explained based on the number of studies carried out across the region and the study design. Apart from that, the differences in prevalence may reflect the type of work performed, national legislation, technical equipment or implementation of control measures, including accessibility and use of protective engineered devices, individual behaviours, education and training [31, 27]. In the Western Pacific region and America, the high prevalence of occupational injuries can be explained based on individual behaviours and socioeconomic inequalities among workers, which influence unsafe acts and make the workers more prone to injuries. This can be supported by a review study done in America about why Americans have a shorter life expectancy and worse health than people in other high-income countries [37]. Also, another review studies mentioned that organizational pressures, schedule delays, certain working habits and ethics such as smoking, use of drugs, drinking, multiple work engagements, and long working hours are the major factors of occupational injuries in Americans [38]. In the African region, some of the countries have inadequate enforcement of health and safety legislation and insufficient safety measures. This is demonstrated by a study done in Tanzania, which revealed that most Tanzanians are not covered by the occupational health and safety law, and only 5% of workers access occupational health services [39]. This situation can make the worker be more vulnerable to suffering from occupational injuries.

Based on the World Bank classification, the findings showed a substantial disparity in the pooled prevalence of occupational injuries. The highest prevalence was in upper-middle-income countries, followed by Africa, and the lowest prevalence was in lower-middle-income countries (Fig. 4). These findings are contrary to the one review titled Comparative Analysis of the Burden of Injury and Illness at Work in Selected Countries and Regions [40], which stated that the developed countries' cases of occupational injuries have been reduced due to tremendous progress in workplace safety and health. In contrast, developing countries due to transition economies experience both high injury and illness risks at work. The variation in occupational injuries in this review may be due to the setting of reviewed studies and the number of workers involved in the studies; also, the differences can be explained based on socioeconomic status among these countries. In LIC, most industrial workers experience poor working conditions where they are exposed to multiple hazards, lack/little training or education about the work they are to perform, little training on OHS, inadequate inspection/monitoring, and low wages. The factors mentioned above are the result of lack of funding and resources for OHS law, lack of political will and governments prioritizing industrial development for its economic growth potential over concern of potential occupational and environmental impacts [41, 42]. All these factors contribute to the occurrence and recurrence of occupational injuries in LIC and also play a role in the disparity of the prevalence of occupational injuries between countries. Another comment is that the papers we found in this study were all focused on individual, personal risk factors. System level risk factors were not mentioned. This might be a part of the cause of the high number of injuries seen in the studies, as systems might be of major importance in preventive work in the industry.

Regarding factors which influence the prevalence of occupational injuries in iron and steel industries, the findings of this review showed that the odds of having occupational injury were 4.06 times higher among workers who did not use PPE compared to those who were using PPE. This finding is higher than the findings from a study done in Ethiopia, which showed that the odds of having occupational injury were 3.01 times higher among workers who did not use PPE compared to their counterparts [34]. Also, our review revealed that the odds of having an occupational injury among workers who had a night shiftwork were 1.65 higher compared to those workers who did not have a night shift work. This finding is supported by the findings from another systematic review and meta-analysis on occupational injuries and work schedule characteristics, which showed that injury risk significantly increased on night [43]. Also a study from Canada showed that night shift work was associated with work injury for both women and men [44]. The findings can be explained basedbyn the fact that humans are biologically programmed to be awake during the day and asleep at night. When this rhythm is shattered, the workers' ability to get enough and good quality sleep suffers. This may leads to fatigue, decreased cognitive functioning, and an increased risk of occupational accidents among night shift workers.

This study tells us that there is a huge disparity between the countries in terms of occupational injuries, where low-income countries and upper middle-income countries seem to be the hotspots of occupational injuries. Hence, we need measures that can adapt to all countries, such as policies based on the economic status of the countries and behavioural change programs to reduce risk to workers worldwide and adjust the disparity between the countries.

Night shiftwork and use of PPE are the predictors of occupational injuries. Therefore, employers should prioritize on the implementation of PPE uses to protect their workers and create a safe work environment.

This study has focused on occupational injuries as a wide search term. In future studies, it might be of interest to focus on specific injuries like burns, amputations and fractures. This angle might give different information about health and safety issues at these workplaces.

### Strengths and limitations

It is an achievement to have conducted the first systematic review and meta-analysis on occupational injuries among workers in iron and steel industries, adopting the QE model for the meta-synthesis of included studies. However, there are some limitations, such as the lack of data from many countries. We obtained data from only 9 countries (Ethiopia, Brazil, India, Poland, Turkey, Saudi Arabia, Singapore, Sweden and Iran), which limits the generalization of our findings. Similarly, some of the studies involved in this review were based on self-reported retrospective data, and the participants may be prone to recall biases. This may account either for over-reporting or under-reporting of occupational injuries among iron and steel industries, we have no knowledge about the effect of this weakness. Including only articles with English language might exclude important studies, absence of fatal injuries will underestimate the results. We also excluded studies from predatory journals, as these journals might lack referee systems, leading to studies with low quality [45]. These conservative exclusion systems used, caused low number of studies: 15. Therefore, the statistical analyses regarding risk factors must be interpreted with caution. Also, this study gives us rough estimates, and other studies are needed for detailed information about the working conditions and risk factors in the industry.

## Conclusions

The pooled prevalence of occupational injuries in the iron and steel industries is 55%, which is considered high in comparison with results from the global prevalence of occupational injuries in general. The results indicate that night work shift and the lack of use of personal protective equipment has a higher impact than other factors in the occurrence of occupational injuries in the iron and steel industries. Personal protective equipment use decreased the occurrence of occupational injuries and night shiftwork increased the rate of occupational injuries occurance in this industry.

## Supplementary Information

Supplementary Material 1: Search key words for the study.

Supplementary Material 2: Quality criteria.

Supplementary Material 3: Forest plot of subgroup analysis of pooled prevalence of occupational injuries based on the WHO region.

Supplementary Material 4: Forest plot of subgroup analysis of pooled prevalence of occupational injuries based on the Data type.

Supplementary Material 5: Forest plot of the odds ratios (OR) with corresponding 95% CIs of studies on the association of marital status and occupational injury.

Supplementary Material 6: Forest plot of the odds ratios (OR) with corresponding 95% CIs of studies on the association of education level and occupational injury.

Supplementary Material 7: Forest plot of the odds ratios (OR) with corresponding 95% CIs of studies on the association of age and occupational injury.

Supplementary Material 8: Forest plot of the odds ratios (OR) with corresponding 95% CIs of studies on the association of safety training and occupational injury.

## Data Availability

Details from the study are given in supplementary files.The datasets used and analysed during the current study are available from the corresponding author on reasonable request.

## Abbreviations

CI: Confidence Interval
LIC: Low-income countries
LMIC: Low- and middle-income countries
MUHAS: Muhimbili University of Health and Allied Sciences
OR: Odds ratio
PPE: Personal protective equipment
QE: Quality effect
SD: Standard deviation
UMIC: Upper middle-income countries
WHO: World Health Organization
