# Supplementary material for: The prevalence of occupational injuries and associated risk factors among workers in iron and steel industries: a systematic review and meta-analysis
Source: BMC Public Health. 2024 Sep 27;24:2602. doi: 10.1186/s12889-024-20111-w (PMC11428562; doi:10.1186/s12889-024-20111-w)
Supplement: Supplementary file 1 — Supplementary Material 1: Search key words for the study. [file 12889_2024_20111_MOESM1_ESM.docx]

**Additional file 1. Electronic search strategy**

| **No** | **Database** | **Search query** |
| --- | --- | --- |
|  | PUBMED | (occupational injury[MeSH Terms]) OR (Occupational accident[MeSH Terms])OR (work related injury[Title/Abstract]) OR (work related accident[Title/Abstract]) OR (Workplace injury[Title/Abstract]) OR (workplace accident[Title/Abstract]) OR (fatal injury[Title/Abstract]) OR (non fatal injury[Title/Abstract]) AND (risk factor[Title/Abstract]) OR (factors[Title/Abstract]) OR (factors associated[Title/Abstract]) OR (predictors[Title/Abstract]) AND (iron industry[Title/Abstract]) OR (steel industry[Title/Abstract]) OR (metal industry[Title/Abstract]) OR (metallurgical industry[Title/Abstract]) Filters: From 1972/1/1-2022/12/31, Full text, Humans, English |
|  | HINARI  -Scholarly peer reviewed | ((injuries) OR (occupational injuries) OR (accidents) OR (occupational accidents)) AND ((risk) OR (risk factors) OR (determinants) OR (predictors) OR (odds ratio)) AND ((iron and steel industries) OR (foundry industries)) Filter: 1972/1/1-2022/12/31 |
|  | Google Scholar | occupational injuries OR Occupational accident AND risk OR risk factors OR odds ratio OR determinants OR predictors "steel industry" OR "Iron industry " OR "foundry industries " Filter: 1972/1/1-2022/12/31 |
|  | EMBASE | #4 AND #5 AND #6  'risk factor*' OR actors OR 'factor* associated' OR predictor* OR 'determinants'/exp OR determinants  'injury'/exp OR injury OR 'accident'/exp OR accident OR 'occupational injury'/exp OR 'occupational injury' OR 'occupational accident'/exp OR 'occupational accident' OR 'work related accident'/exp OR 'work related accident' OR 'work related injury'/exp OR 'work related injury' OR 'workplace injury' OR 'fatal injur*' OR 'non fatal injur*'  ('metallurgy'/exp OR metallurgy OR 'steel industry'/exp OR 'steel industry' OR 'iron industry'/exp OR 'iron industry' OR 'metal industry'/exp OR 'metal industry' OR 'steel'/exp OR steel) AND ('industry'/exp OR industry) OR 'metal industry'/exp OR 'metal industry'  'metallurgy'/exp OR metallurgy |
