# Supplementary material for: The prevalence of occupational injuries and associated risk factors among workers in iron and steel industries: a systematic review and meta-analysis
Source: BMC Public Health. 2024 Sep 27;24:2602. doi: 10.1186/s12889-024-20111-w (PMC11428562; doi:10.1186/s12889-024-20111-w)
Supplement: Supplementary file 2 — Supplementary Material 2: Quality criteria. [file 12889_2024_20111_MOESM2_ESM.docx]

Additional file 2. Quality assessment results

| **SN** | **Author year** | **Quality criteria score** | | | | | | | | | |
| --- | --- | --- | --- | --- | --- | --- | --- | --- | --- | --- | --- |
|  |  | **A** | **B** | **C** | **D** | **E** | **F** | **G** | **H** | **I** | **Total** |
|  | Asadi Z. | 1 | 0 | 1 | 1 | 0 | 1 | 1 | 1 | 0 | 6 |
|  | Bahrami A. | 1 | 1 | 1 | 0 | 0 | 0 | 0 | 0 | 1 | 4 |
|  | Ballal SG. | 1 | 1 | 1 | 1 | 0 | 0 | 1 | 1 | 0 | 6 |
|  | Benti A. | 1 | 1 | 1 | 0 | 0 | 1 | 1 | 1 | 1 | 7 |
|  | Berhan E. | 1 | 1 | 1 | 0 | 0 | 1 | 0 | 1 | 1 | 6 |
|  | Bylund PO | 1 | 1 | 1 | 1 | 0 | 0 | 1 | 1 | 0 | 6 |
|  | Dell T. | 1 | 1 | 0 | 0 | 0 | 0 | 1 | 1 | 0 | 4 |
|  | Durmaz S. | 1 | 0 | 0 | 0 | 0 | 1 | 0 | 1 | 1 | 4 |
|  | Gonçalves SB. | 1 | 1 | 1 | 1 | 0 | 0 | 1 | 1 | 0 | 6 |
|  | Gulhan B. | 1 | 0 | 1 | 0 | 0 | 0 | 1 | 1 | 1 | 5 |
|  | Jafari etal. | 1 | 0 | 1 | 1 | 0 | 0 | 1 | 0 | 0 | 4 |
|  | Kilfe M. | 1 | 1 | 1 | 0 | 0 | 1 | 0 | 1 | 1 | 6 |
|  | Kumar SG. | 1 | 0 | 1 | 1 | 0 | 1 | 1 | 1 | 1 | 7 |
|  | Manjunatha R. | 1 | 1 | 1 | 0 | 0 | 0 | 0 | 0 | 1 | 4 |
|  | Mazaheri MA. | 1 | 1 | 1 | 1 | 0 | 0 | 1 | 0 | 0 | 5 |
|  | Ogiński A. | 1 | 1 | 1 | 1 | 0 | 0 | 1 | 1 | 0 | 6 |
|  | Ong CN. | 1 | 1 | 1 | 1 | 0 | 0 | 1 | 1 | 0 | 6 |
|  | Rajak R. | 1 | 1 | 1 | 1 | 0 | 1 | 1 | 1 | 1 | 8 |
|  | Schoemaker MJ. | 1 | 1 | 1 | 1 | 0 | 1 | 1 | 1 | 0 | 7 |
|  | Sime A. | 1 | 1 | 1 | 0 | 0 | 1 | 1 | 1 | 1 | 7 |
| **Average** | | | | | | | | | | | 5.75 |

Note: A -– appropriateness of the way used to select study participants, B – adequateness of sample size, C – description of study subjects and settings, D – Standard criteria used for measurement of condition, E–identification of confounding factors, F– Strategies to deal with confounding factors, G- Reliability way of measuring the outcome H – appropriateness of statistical tests used in data analysis and I – adequateness of response rate.
