# Supplementary figures and images for: The prevalence of occupational injuries and associated risk factors among workers in iron and steel industries: a systematic review and meta-analysis
Source: BMC Public Health. 2024 Sep 27;24:2602. doi: 10.1186/s12889-024-20111-w (PMC11428562; doi:10.1186/s12889-024-20111-w)

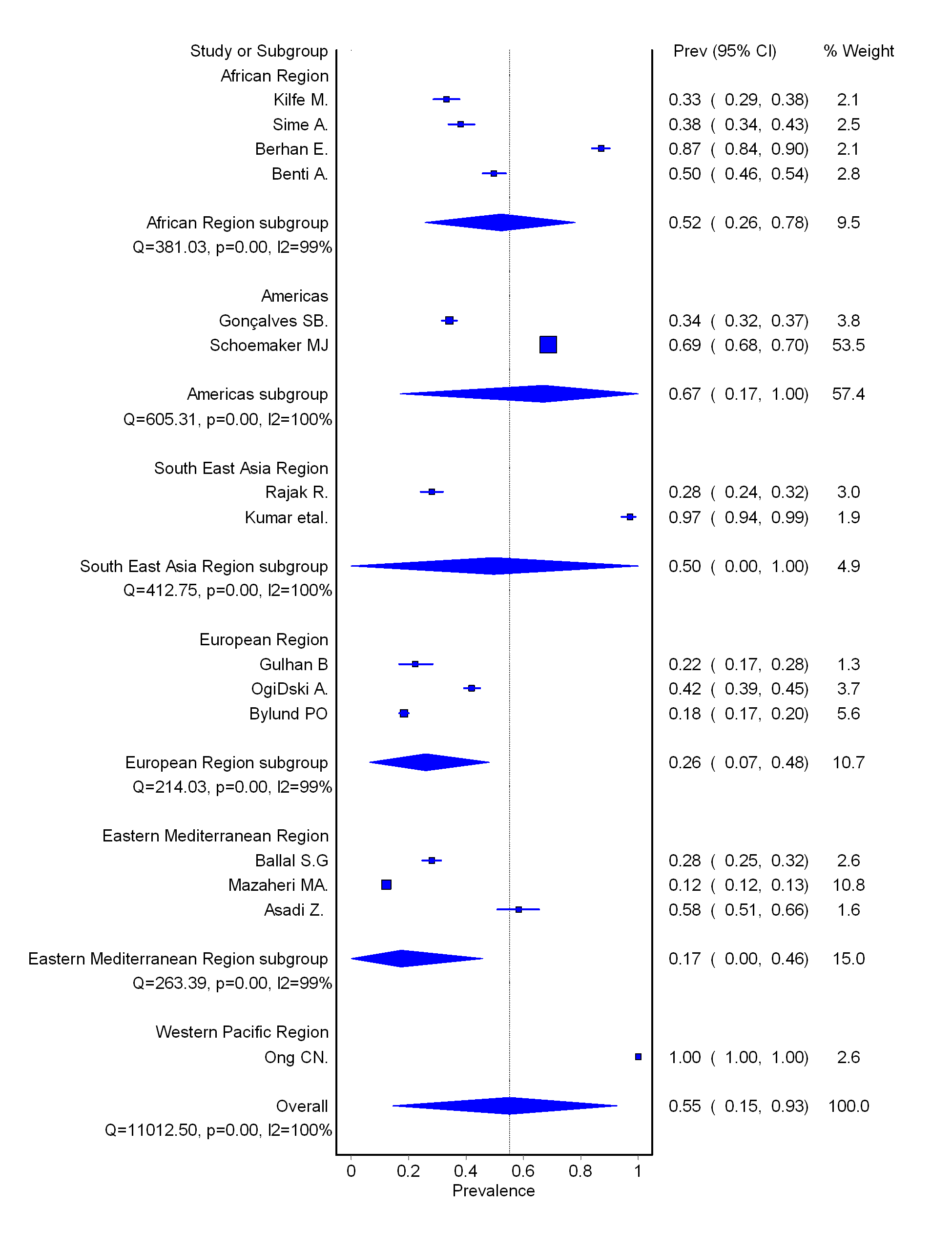

Supplement: Supplementary file 3 — Supplementary Material 3: Forest plot of subgroup analysis of pooled prevalence of occupational injuries based on the WHO region. [file 12889_2024_20111_MOESM3_ESM.tif]

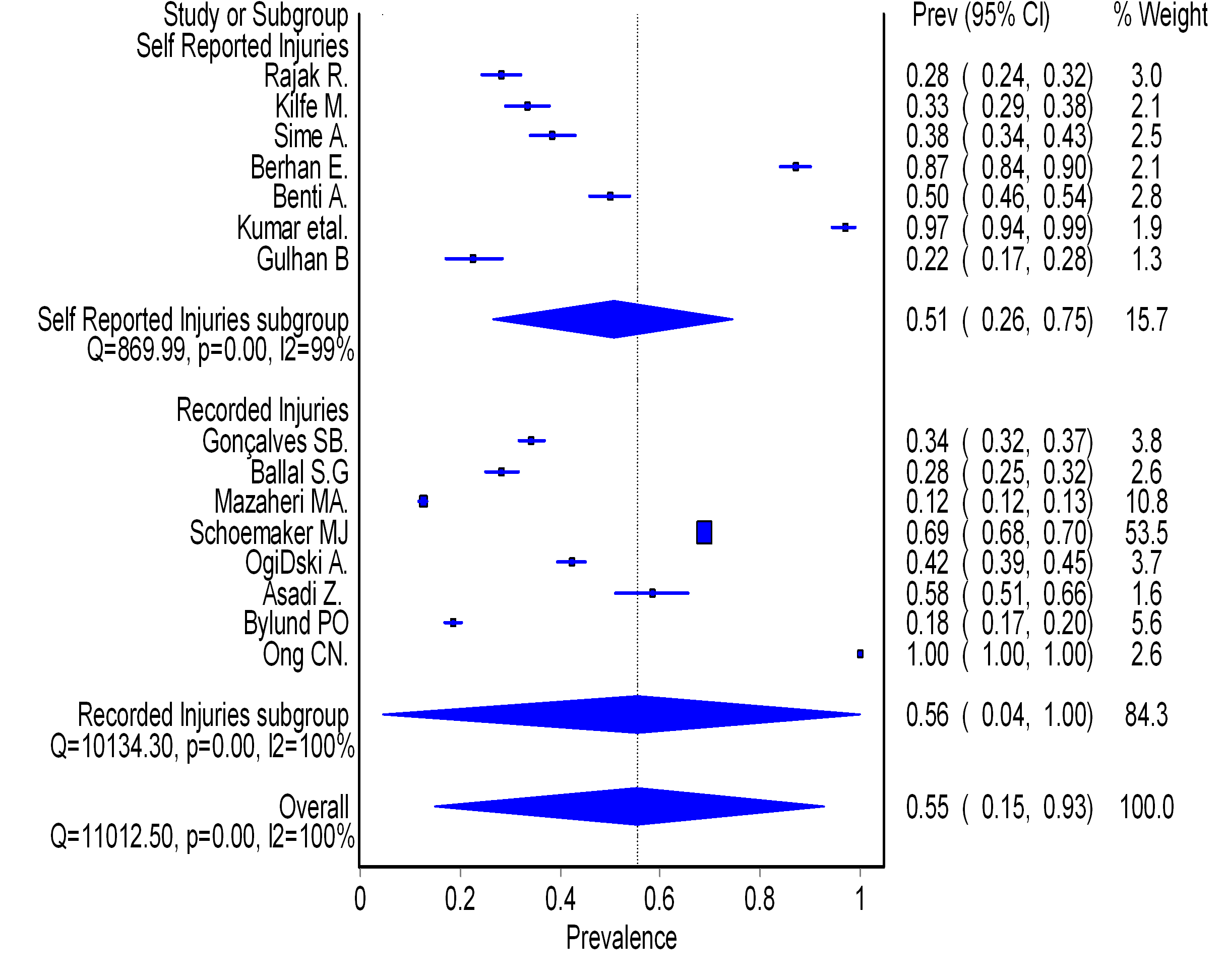

Supplement: Supplementary file 4 — Supplementary Material 4: Forest plot of subgroup analysis of pooled prevalence of occupational injuries based on the Data type. [file 12889_2024_20111_MOESM4_ESM.tif]

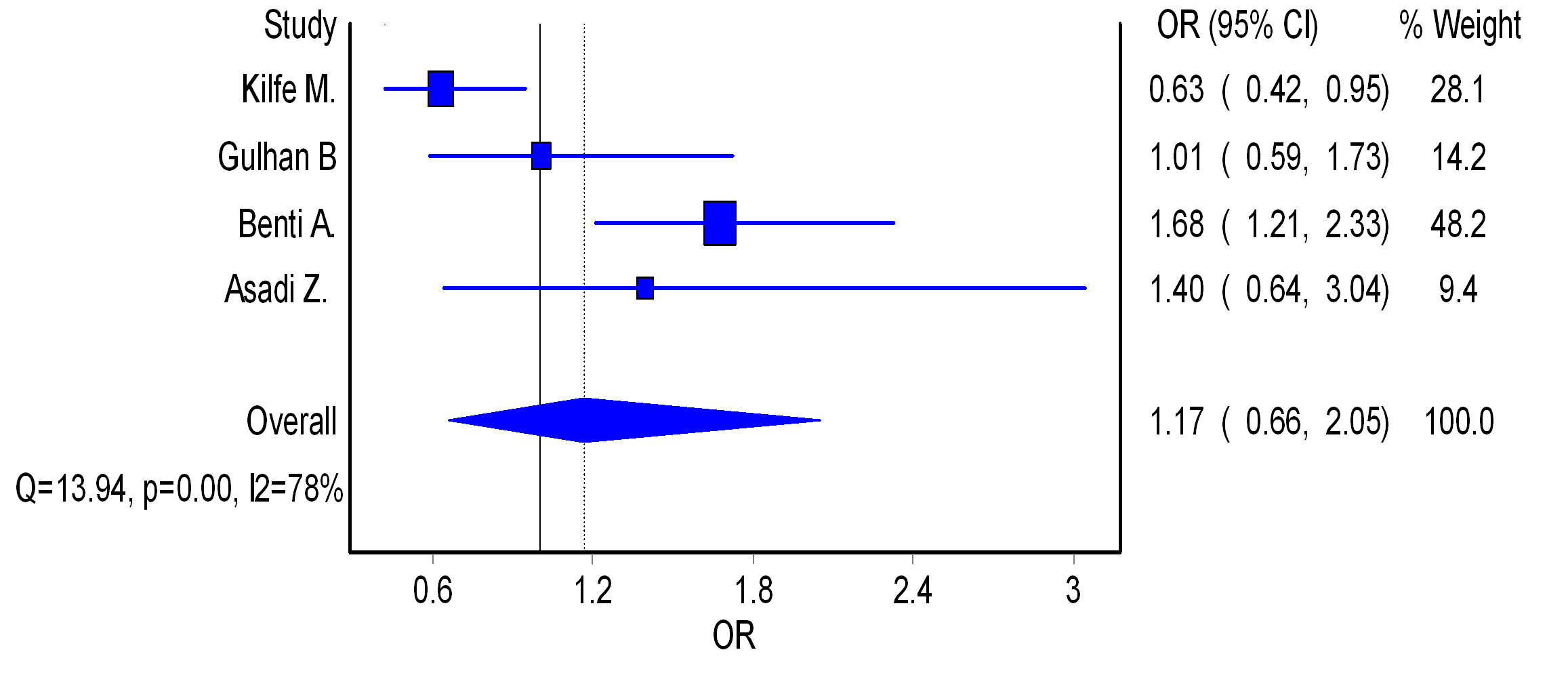

Supplement: Supplementary file 5 — Supplementary Material 5: Forest plot of the odds ratios (OR) with corresponding 95% CIs of studies on the association of marital status and occupational injury. [file 12889_2024_20111_MOESM5_ESM.tif]

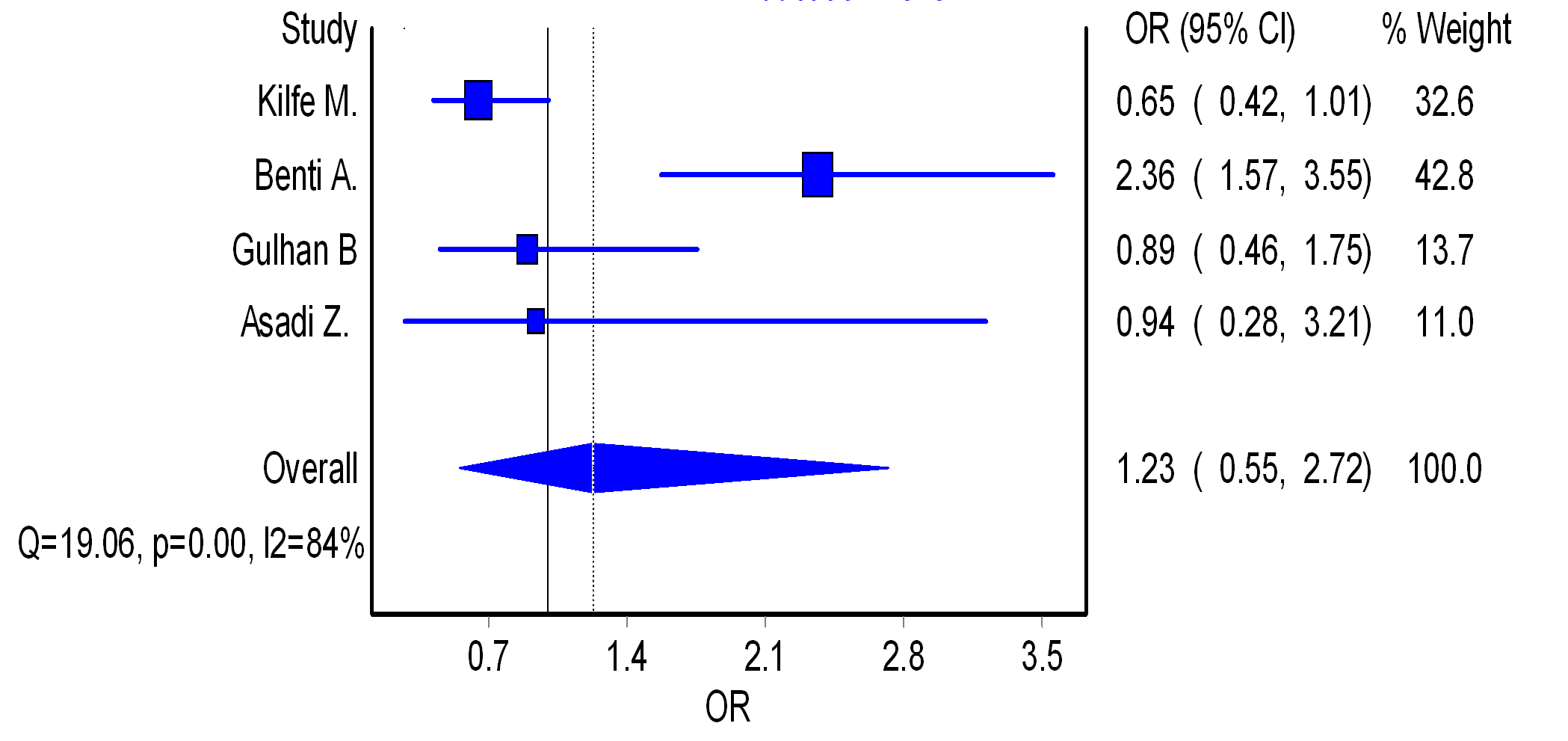

Supplement: Supplementary file 6 — Supplementary Material 6: Forest plot of the odds ratios (OR) with corresponding 95% CIs of studies on the association of education level and occupational injury. [file 12889_2024_20111_MOESM6_ESM.tif]

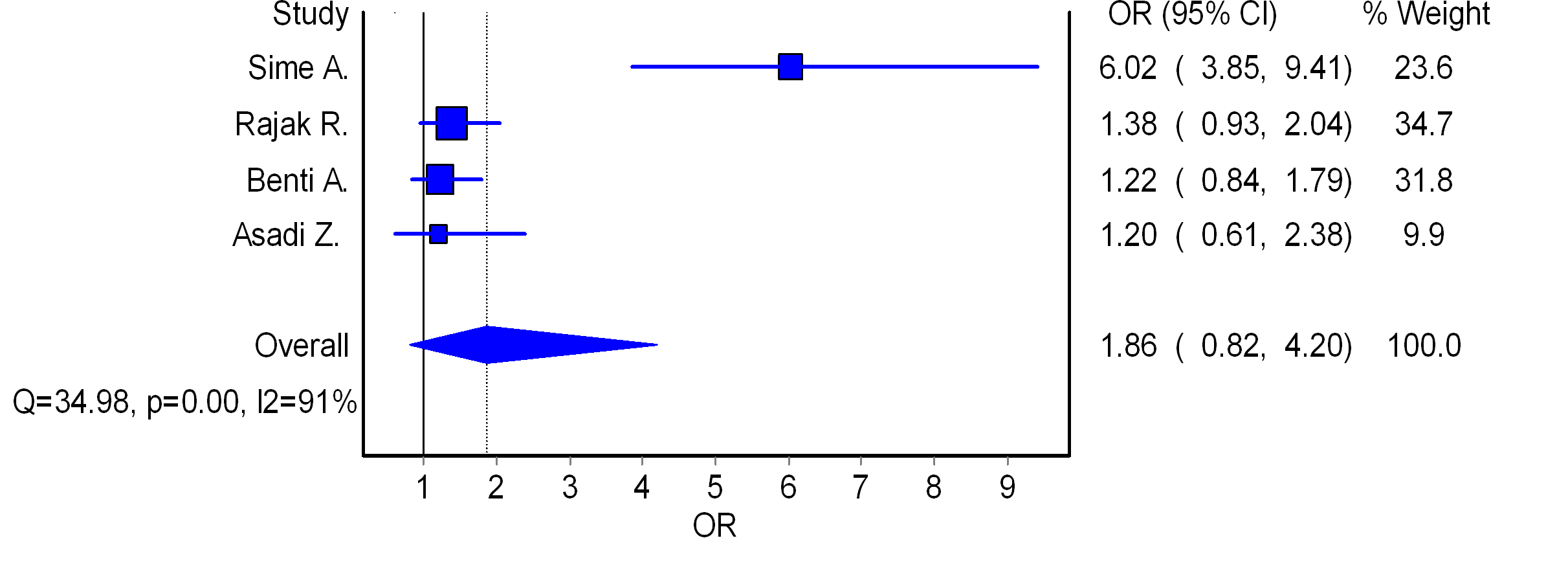

Supplement: Supplementary file 8 — Supplementary Material 8: Forest plot of the odds ratios (OR) with corresponding 95% CIs of studies on the association of safety training and occupational injury. [file 12889_2024_20111_MOESM8_ESM.tif]
